# A review of research advances in the modulation of olfactory receptors for COPD inflammation and airway remodeling

**DOI:** 10.3389/fimmu.2025.1612165

**Published:** 2025-07-04

**Authors:** Zhihao Zhu, Daohui Gong, Yue Chen, Ming Yuan, Hang Qian, Binfeng He, Guansong Wang

**Affiliations:** ^1^ Institute of Respiratory Diseases, Xinqiao Hospital, Third Military Medical University, Chongqing, China; ^2^ Department of General Practice, Xinqiao Hospital, Third Military Medical University, Chongqing, China

**Keywords:** olfactory receptors, chronic obstructive pulmonary disease, chronic inflammation, airway remodeling, therapeutic targets

## Abstract

Chronic obstructive pulmonary disease (COPD), as the third leading cause of global mortality, presents complex pathological mechanisms and imposes a substantial health burden. Emerging evidence reveals that olfactory receptors (ORs), traditionally associated with odor detection, exhibit non-canonical regulatory functions in COPD pathogenesis. This review systematically explores ORs’ multidimensional roles: environmental triggers activate specific ORs in specific cells, initiating chronic inflammatory cascades. Persistent inflammation drives irreversible airway remodeling through smooth muscle proliferation and extracellular matrix reorganization. Preclinical and clinical studies demonstrate that OR agonists/antagonists modulate the inflammation-remodeling axis to influence pulmonary function, though their pleiotropic effects complicate therapeutic targeting. The cell type-specific expression patterns and diverse ligand profiles of ORs create unique opportunities for precision interventions, while posing challenges in tissue delivery and receptor efficacy optimization. Future investigations should integrate single-cell omics and artificial intelligence to elucidate OR-mediated dynamic networks, downstream signaling pathways, and their interplay with microbiome-gut-lung axis regulation. This review not only advances our understanding of OR biology in respiratory diseases but also proposes a novel theoretical framework for developing OR-based diagnostic and therapeutic strategies in the early management of COPD.

## 1 Introduction

COPD is a progressive respiratory disorder marked by irreversible airflow limitation. COPD affects over 300 million people globally and imposes a heavy burden on healthcare systems due to its high morbidity and mortality. COPD not only severely impairs patients’ quality of life but also places a heavy economic and social burden on society (1, 2). Although cigarette smoke and air pollutants are well-established risk factors for COPD, its complex pathogenesis remains incompletely understood. Interestingly, recent researches reveal that ORs may play an unexpected role in the occurrence and development of COPD.

ORs are primarily known for their role in detecting odorants in the olfactory epithelium (3). In 1991, Linda Buck and Richard Axel discovered a large gene family encoding ORs, for which they were awarded the Nobel Prize in Physiology or Medicine in 2004 (4). Initially, ORs are thought to be exclusive to the olfactory system, but emerging evidence indicates that ORs are also expressed in various extra-nasal tissues, including lungs (**Table 1**), where they may be involved in diverse physiological and pathological processes. Among these, certain ORs have been functionally confirmed through knockout or knockdown experiments. OR51E2 was verified via knockout experiments (5); OR2W3, OR51B5, and OR2AT4 were validated through knockdown experiments (6–8). Although OR51B5 and OR2AT4 were confirmed in other cell types, they have similar functions in the lungs.

**Table 1:** Olfactory receptors in lung tissue.

Cell type	Olfactory receptor	Agonist	Function	Validation Method	Refs
HASMCs	OR2W3	Monoterpene nerol	Relax smooth muscle cells	Knockdown	(6)
	OR51E2	Acetate, propionate	Inhibit cytoskeletal remodeling and cell proliferation	Knockout	(5)
	OR2AG1	Amyl butyrate	Inhibit smooth muscle cell contraction		(19)
	OR1D2	Bourgeonal	Promote smooth muscle cell contraction		(19)
Human bronchial epithelial cells	OR51B5	Farnesol, isononyl alcohol	Reduce cell viability and induce the release of IL-8 and IL-6	Knockdown (Hs68 cells)	(7, 39)
	OR1G1	Nonanal, γ-Decalactone	Induce the release of IL - 8 and IL - 6		(39)
	OR2AT4	Brahmanol, sandalore	Enhance wound healing	Knockdown (Human keratinocytes)	(8, 40)
	OR2J3	Cinnamaldehyde, helional	Inhibit IL-8 secretion and wound healing		(40)
Human primary alveolar macrophages	OR2AT4	Sandalore, brahmanol	Decrease the phagocytic activity of alveolar macrophages and reduce the secretion of pro-inflammatory factors such as CXCL-8 and IL-6		(18)
	OR1A2	Citronellal			
PNECs	OR2W1	Nonanal, citronellal	Decrease the level of 5-HT in PNECs and promote the release of CGRP		(45)
	OR2F1	Nonanal, citronellal			

Current COPD management primarily relies on inhaled corticosteroids and bronchodilators, which fail to halt disease progression. Notably, steroid resistance frequently occurs in COPD patients despite inflammation being a pivotal initiating mechanism. This paradox drives intensive research on molecular targets including IL-5/IL-5R, IL-4(R)/IL-13, TSLP, IL-33, and ST2 for precision therapies (9, 10). Two novel agents—Ensifentrine (11) (a phosphodiesterase inhibitor) and Dupilumab (12) (an IL-4 receptor antagonist)—have recently been incorporated into the 2025 Global Initiative for Chronic Obstructive Lung Disease (GOLD) recommendations for preliminary clinical application.

**Figure 1:**
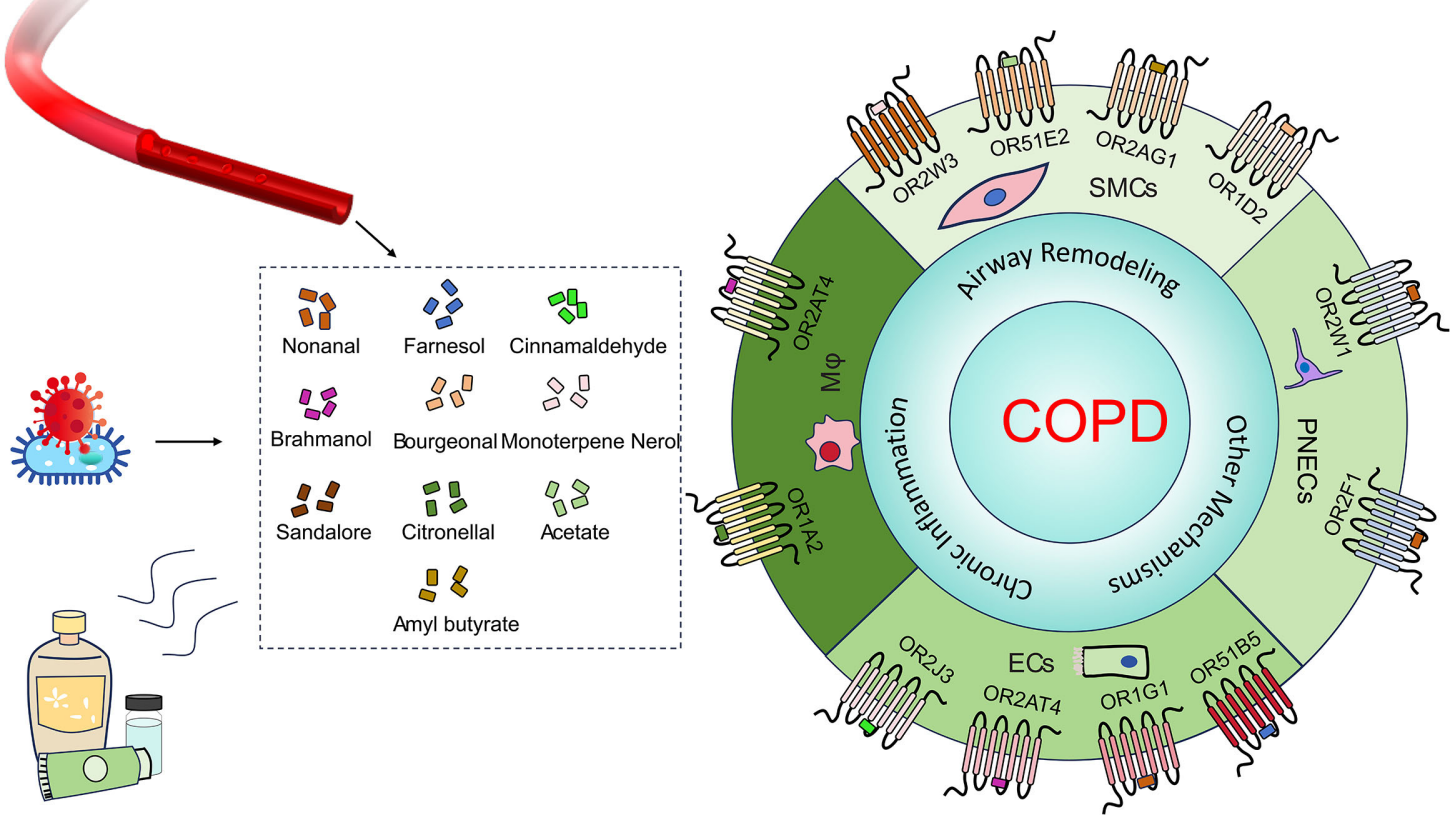
Ligands and ORs in COPD. Common olfactory receptor ligands (e.g., nonanal, farnesol and citronellal) originate from fragrance products, industrial solvents, microbial metabolites, endogenous sources and so on. These ligands act on cell type-specific ORs (e.g., OR2AT4, OR51E2, OR2W1 and OR1A2) expressed in airway ECs, airway smooth muscle cells (SMCs), macrophages (Mφ), and PNECs within lung tissue. They directly influence COPD through mechanisms involving chronic inflammation and airway remodeling.

Nevertheless, developing multi-mechanism therapeutic strategies remains imperative, particularly targeting concurrent inflammation and airway remodeling. Intriguingly, accumulating evidence positions ORs as dual regulators of inflammatory responses and structural alterations in COPD pathogenesis (**Figure 1**). OR-targeted approaches hold promise as novel therapeutic targets capable of exerting synergistic effects with current treatments. This may be particularly beneficial for odorant-sensitive patient subgroups, potentially enabling better symptom control and prevention of disease progression. Notably, given the subset of COPD patients exhibiting corticosteroid resistance, OR modulation could offer a valuable alternative therapeutic avenue. Therefore, further investigation into OR-mediated molecular networks could unveil novel biomarkers for early diagnosis and inspire innovative therapeutic approaches against this recalcitrant disease.

## 2 Basic concepts and pathological mechanisms of COPD

### 2.1 Definition and classification of COPD

COPD is defined as a common, preventable, and treatable disease characterized by persistent respiratory symptoms and airflow limitation due to airway and/or alveolar abnormalities, usually caused by significant exposure to noxious particles or gases (13). According to the Global Initiative for Chronic Obstructive Lung Disease (GOLD) criteria, the post-bronchodilator FEV1/FVC < 0.70 is used to diagnose airflow limitation, which is a key feature of COPD. In addition to pulmonary function tests, imaging studies like chest computed tomography scans are valuable for assessing lung structure changes and disease severity (14). Besides, COPD can be classified into two main phenotypes: chronic bronchitis and emphysema. Chronic bronchitis is characterized by persistent cough and sputum production, while emphysema is manifested as alveolar destruction and airflow limitation. Early identification and intervention of COPD are crucial for improving patients’ quality of life and prognosis. Figuring out how to achieve these is a key aspect that we need to study intensively in the next step.

### 2.2 Pathogenesis of COPD

The pathogenesis of COPD is multifactorial and involves complicated interactions between environmental factors, immune responses, and genetic susceptibility (1). Environmental factors like smoking, biomass fuel exposure, occupational dust, chemical exposure, and air pollution are major precipitating factors for COPD development. These noxious agents cause chronic inflammation and oxidative stress in the lungs, leading to tissue damage and remodeling. The immune response in COPD is dysregulated, with the activation of multiple immune cells, such as neutrophils, macrophages, lymphocytes, and eosinophils (15). These cells secrete a plethora of inflammatory mediators and cytokines, including interleukin-6 (IL-6), interleukin-8 (IL-8), tumor necrosis factor-α (TNF-α), and chemokines, which can recruit more immune cells to the lungs, perpetuating the inflammatory process and contributing to airway remodeling and lung tissue destruction. Genetic factors also play a role in COPD susceptibility. Mutations in the SERPINA1 gene, which encodes alpha-1 antitrypsin, are the most well-characterized genetic risk factors, leading to hereditary alpha-1 antitrypsin deficiency (AATD) (16). However, other genetic variations associated with COPD, such as those related to alpha1-antichymotrypsin, alpha2-macroglobulin, and vitamin D-binding protein, are still under investigation (17). Additionally, multiple pathogenic mechanisms—including recurrent infections, mucus hypersecretion, and microbial dysbiosis—contribute differentially to COPD development. Notably, chronic airway inflammation and subsequent tissue remodeling triggered by diverse factors remain central to disease pathogenesis.

While the pathogenesis of COPD is well-established to involve chronic inflammation, oxidative stress, and airway remodeling, current therapeutic strategies remain insufficient to halt disease progression. Intriguingly, emerging evidence suggests that ORs may directly participate in COPD pathogenesis by regulating immune cell activation, inflammatory cytokine release, and smooth muscle dynamics. For instance, activation of OR2AT4 in alveolar macrophages reduces IL-6 and C-X-C motif chemokine ligand 8 (CXCL-8) secretion, thereby highlighting ORs’ anti-inflammatory role in the disease microenvironment (18); activation of OR2W3 in airway smooth muscle cells promotes relaxation through Ca²^+^ influx (6). Conversely, aberrant activation of OR1D2 in airway smooth muscle cells enhances contraction via Ca²^+^ influx, potentially exacerbating airflow limitation (19). These findings suggest that ORs extend beyond their canonical role in odor detection, forming a dynamic interaction network with core COPD pathways through non-canonical signaling.

## 3 Biological characteristics of olfactory receptors

### 3.1 Structure and function of olfactory receptors

Olfactory receptors belong to the superfamily of G protein-coupled receptors (GPCRs), which are integral membrane proteins with seven transmembrane helices. In mammals, the OR gene family is one of the largest GPCR families, with approximately 370 genes in humans (20). ORs are predominantly expressed in the olfactory epithelium, where they function as chemo-sensors to detect a vast array of odorant molecules. The structural diversity of olfactory receptors enables them to recognize and discriminate thousands of different odorant molecules, thus playing a vital role in olfactory perception. Their ligands can be classified into several categories of chemical substances based on their chemical structures, including alcohols, aldehydes, acids, and esters (21).

### 3.2 Role and general mechanism of olfactory receptors in other systems

Beyond the olfactory system, ORs have been detected in various non-olfactory tissues, including the respiratory tract, digestive system, skin, and immune cells (22–25). In these tissues, ORs are involved in a wide range of physiological and pathological processes. For example, in macrophages, ORs can modulate the inflammatory response (26). Activation of certain ORs in macrophages has been shown to exacerbate atherosclerosis (25) and promote tumor progression (27). In the skin, ORs are involved in hair growth (28) and wound healing (8). In the digestive system, they participate in glucose metabolism (29).

Generally speaking, how do olfactory receptors function? The binding of ligands to ORs activates a G-protein, typically Gαolf, which in turn activates adenylate cyclase (AC), leading to an increase in intracellular cyclic adenosine monophosphate (cAMP) levels. cAMP then activates cyclic nucleotide-gated cation channels (CNG channels), causing an influx of calcium ions (Ca^2+^) into the cells. The Ca^2+^ influx triggers a series of downstream signaling pathways (18, 25). The signal transduction mechanisms of ORs in non-olfactory tissues are similar to those in the olfactory epithelium but also have different downstream effects depending on the cell types. In addition to the cAMP-dependent pathway, ORs can also activate other signaling cascades, such as the phosphatidylinositol 3-kinase (PI3K)-Akt pathway and the mitogen-activated protein kinase (MAPK) pathway, which regulate cell survival, proliferation, migration, and differentiation (8, 30).

### 3.3 Research progress in the molecular biology of olfactory receptors

In recent years, remarkable progress has been made in molecular biology research on ORs. Through genomic and bioinformatics approaches, researchers have identified and annotated numerous OR genes, particularly in vertebrates. These studies have provided critical insights into the evolution and functions of ORs (31). Structural biology techniques, such as X-ray crystallography and cryo-electron microscopy, are instrumental in determining the three-dimensional structures of ORs bound to their ligands. These structural studies have elucidated the molecular mechanisms of ligand-receptor interactions, which are essential for developing novel drugs targeting ORs (32). Furthermore, functional genomics approaches, including gene knockout and overexpression studies in animal models, have helped clarify the physiological roles of specific ORs (25). These findings have laid a solid foundation for understanding the role of ORs in health and disease and for advancing targeted therapies.

## 4 Olfactory receptors and COPD

### 4.1 Olfactory receptor ligands in the respiratory tract

In modern daily life, individuals are exposed to a diverse range of odorants from various sources, many of which are ligands for ORs (**Table 2**). Notably, patients with COPD have been found to exhibit elevated levels of volatile organic compounds (VOCs) in their exhaled breath compared to healthy individuals. These VOCs, such as benzaldehyde, isoprene, hexanal and nonanal, are promising candidates for COPD biomarkers. Specifically, increased levels of nonanal are associated with smoking behavior (33, 34).

**Table 2:** Main sources of olfactory receptor ligands.

Source	Ligand	Ref./Data sources
Cigarette/e-cigarette smoke	Butyraldehyde, decanal, hexanal, nonanal, cinnamaldehyde, vanillin, ethanol, benzaldehyde	(35, 36, 99–101)
Air	Acetate, methanethiol, hexanal, benzaldehyde, heptanal, benzothiazole, ethanol, isopropylbenzene, pentanal	(37, 38)
Aromatic products	Cinnamaldehyde, citral, sandalwood, farnesol, lyral	https://china.guidechem.com/datacenter/hzp.html, (74)
Gut microbiota	Acetate, propionate	(42)
Lung microbiota	Heptane, methylated cycloalkanes	(43)
Body metabolism	Hexanal, octanal, farnesol	(44)

As major risk factors for COPD, cigarette smoke and air pollution contain various ligands for ORs, including nonanal, cinnamaldehyde and hexanal (35–38). Nonanal and cinnamaldehyde have been shown to promote the secretion of inflammatory cytokines, such as IL-6 and IL-8 (39, 40), which are key mediators in the pathogenesis of COPD. Aromatic products, including cosmetics, can release odorants that may interact with ORs in the respiratory tract. Although the effects of these products on the respiratory system are not fully understood, skin contact-induced inflammatory reactions suggest potential influence on the respiratory tract (41). As is known to all, the respiratory tract and the gut are colonized by microbiota. Their metabolites, such as acetate and propionate, also act as ligands for ORs (42, 43). Additionally, endogenous metabolism, particularly lipid peroxidation, generates various aldehydes, including hexanal, octanal, and farnesol, which can also bind to ORs in the respiratory tract (44). These ligands can influence the activity of ORs in airway cells, potentially influencing COPD development and progression through mechanisms such as chronic inflammation and airway remodeling.

### 4.2 Clinical research on olfactory receptors and obstructive lung diseases

In recent years, research into the role of ORs in obstructive lung diseases, particularly COPD, has gained momentum. Jürgen Knobloch’s research group successfully isolated and cultured primary bronchial epithelial cells from non-COPD patients, demonstrating that these cells functionally express four specific ORs: OR51B5, OR1G1, OR2AT4, and OR2J3 (39, 40). Farnesol, isononyl alcohol and nonanal can activate these ORs respectively, and influence the secretion of inflammatory cytokines IL-6 and IL-8, which suggests that ORs have a potential role in airway inflammation. Human airway smooth muscle cells (HASMCs) play a crucial role in airway remodeling, a hallmark feature of COPD. Additionally, several research teams have isolated HASMCs and identified the expression of various ORs, including OR2W3, OR51E2, OR2AG1, and OR1D2 (5, 6, 19).

Weidinger D et al. conducted a study on alveolar macrophages isolated from patients with COPD, asthma, or chronic bronchitis, and demonstrated significant expression of olfactory receptors OR2AT4 and OR1A2 (18). Activation of these receptors was found to reduce the secretion of pro-inflammatory cytokines and inhibit inflammation induced by lipopolysaccharide (LPS), lipoteichoic acid (LTA), or peptidoglycan (PGN). Additionally, pulmonary neuroendocrine cells (PNECs), despite their low abundance in the lung, have been shown to increase in number in COPD patients, with a concurrent upregulation of OR2W1 receptor expression in these cells. This finding suggests a potential role of OR2W1 in neurotransmitter secretion and airway regulation (45).

While these findings are promising, current clinical research still has several limitations. The experimental designs are often not rigorous enough, with many studies relying solely on LPS stimulation without considering the combined effects of cigarette smoke extract (CSE) and other relevant factors. Besides, sample sizes are relatively small, and there is a lack of multi-center randomized controlled trials (RCTs). These limitations restrict the generalizability and reliability of the results, underscoring the need for more comprehensive and well-designed studies.

### 4.3 Related research in animal models

In COPD animal models, research on ORs is still in its early stage. Nevertheless, some studies have demonstrated that treatment with the OR agonist farnesol can protect the lungs from cigarette smoke-induced damage in COPD-like animal models (46). Farnesol exerts its protective effects by inhibiting pulmonary inflammation, reducing oxidative stress, and enhancing antioxidant capacity. Li, Tay, and colleagues utilized microarray and q-PCR to evaluate the expression of ORs in airway and pulmonary macrophages of mice treated with IFN-γ, LPS, or IFN-γ/LPS. Their findings identified a group of ORs (OR65, OR272, OR352, OR446, OR568, OR622, OR657, and OR1014) that were expressed in mouse airway and pulmonary macrophages and were significantly upregulated under IFN-γ/LPS treatment. Furthermore, stimulation with octanal (an agonist of ORs) promotes the release of monocyte chemoattractant protein-1 (MCP-1) and enhances macrophage chemotaxis, leading to increased immune cell infiltration and exacerbated local inflammation (47).

Animal model studies have demonstrated the potential of ORs in the pathogenesis of COPD-like conditions and have identified promising therapeutic targets. However, further research is required to fully harness these findings and translate them into clinical applications.

### 4.4 Olfactory receptors in airway inflammatory regulation

#### 4.4.1 Multicellular inflammatory microenvironment in airway inflammation pathogenesis

**Figure 2:**
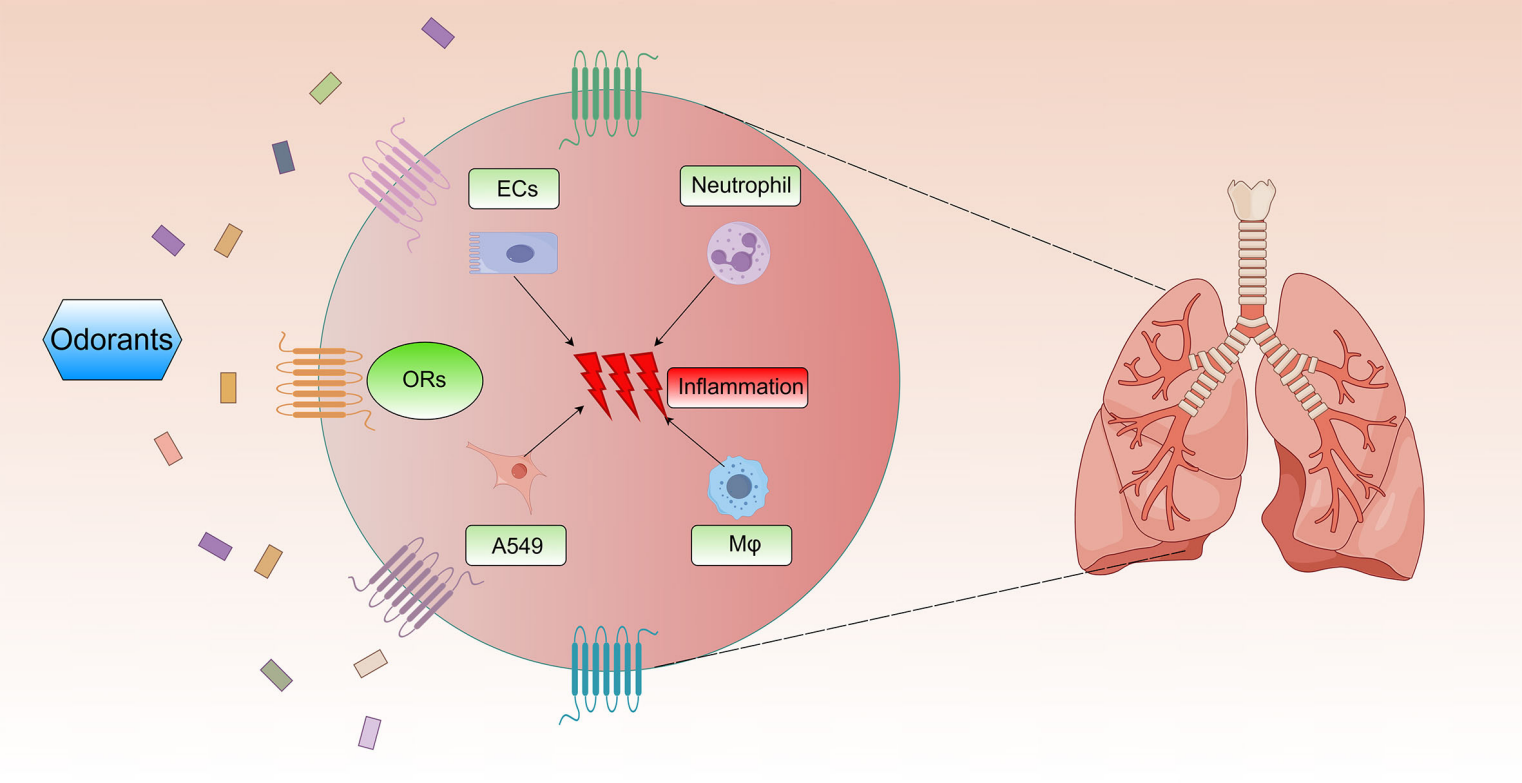
Effects of ORs on chronic inflammation in COPD. Multiple odorants activate ORs on the surface of airway epithelial cells, neutrophils, A549 cells, and alveolar macrophages. Then, they can trigger pulmonary inflammatory injury in COPD.

The chronic inflammatory microenvironment in COPD emerges through coordinated interactions between immune cells and structural cells (**Figure 2**). This pathological process centers on persistent airway and parenchymal inflammation that persists post-smoking cessation (48), directly correlating with disease progression. Macrophages act as central orchestrators, initiating inflammatory cascades via pro-inflammatory mediators (IL-8, IL-6) (49). Neutrophils subsequently infiltrate lung tissue, inducing direct damage through elastase and reactive oxygen species while establishing self-perpetuating inflammatory loops (50). Airway epithelial cells simultaneously function as physical barriers and active contributors through inflammatory cytokine secretion. Crucially, these cellular components operate through interconnected cytokine networks that establish self-reinforcing feedback loops. Such multicellular crosstalk drives progressive airway inflammation, ultimately manifesting as irreversible structural changes characterized by small airway fibrosis and pulmonary emphysema.

COPD pathogenesis predominantly features neutrophil-driven inflammation (51), with coordinated involvement of macrophages, NK cells, and B cells in sustaining chronic airway inflammation (52–54). Environmental exposures (cigarette/e-cigarette smoke, air pollutants) disrupt pulmonary immune homeostasis, exacerbating disease severity through amplified inflammatory responses (55). Over 50 types of ORs are expressed on the surface of neutrophils (56). Activation of these ORs by components of the butter aroma complex (diacetyl, butanoic acid, and δ-decalactone) promotes cAMP production and induces neutrophil chemotaxis (57). Furthermore, in alveolar macrophages, the activation of OR2AT4 and OR1A2 by sandalore and citronellal reduces phagocytic activity and the secretion of pro-inflammatory cytokines, such as CXCL-8 and IL-6 (18). Additionally, cinnamaldehyde, a major component of e-cigarettes, can inhibit the phagocytic function and cell viability of neutrophils and alveolar macrophages (35), further emphasizing the role of ORs in modulating immune cell function in COPD.

Meanwhile, airway epithelial cells serve as the first line of defense against external insults, constantly exposed to cigarette smoke, pollutants, pathogens, and so on. Disruption of airway epithelial barrier integrity by these stimuli permits the entry of harmful substances, initiating an inflammatory response. Exposure of tracheal epithelial cells to farnesol, isononyl alcohol or nonanal—components of various environmental pollutants—activates ORs such as OR51B5 or OR1G1, leading to the release of pro-inflammatory cytokines IL-6 and IL-8 (39). These cytokines play a critical role in the pathogenesis of COPD, with elevated IL-8 levels being strongly associated with acute exacerbations of COPD (AE-COPD) (58). However, the effect of OR51B5 on cell viability appears to be cell-type-dependent: while it accelerates the regeneration rate of the HaCaT cell and keratinocyte monolayers (59), it reduces viability in bronchial epithelial cells (39). This differential effect suggests that OR51B5 regulates cell behavior through complex mechanisms that warrant further investigation.

Disruption of alveolar structure is another hallmark of COPD pathophysiology. In COPD patients, alveoli undergo significant destruction and atrophy, causing a reduction in alveolar surface area and impaired gas exchange efficiency. These structural changes are primarily driven by chronic inflammation and oxidative stress, which induce apoptosis and fibrosis of alveolar walls. Smoking and air pollution are major external factors contributing to these processes. Alveolar type II epithelial (ATII) cells play a crucial role in this context, as they exhibit increased expression of inflammation-related genes, such as IL-6, CXCL1, and CXCL8, compared to non-smokers. When exposed to cigarette smoke extract (CSE), A549 cells will release plenty of inflammatory factors (60). However, the response of primary ATII cells to CSE stimulation is variable: IL-8 secretion increases in some samples but decreases in others. Even at higher CSE concentrations (e.g., 5%), the secretion of IL-8, MCP-1, and growth-regulated oncogene-α (GRO-α) diminishes (61). Interestingly, as a component of CSE and an agonist of OR51B5, farnesol can also enhance the secretion of IL-8 and IL-6 in A549 cells and inhibit cell activity (39). Conversely, in animal experiments, farnesol increases glutathione levels, reduces hydrogen peroxide content, alleviates CSE-induced lung inflammation, and preserves lung function (46, 62). Of course, differences exist between A549 cells and ATII cells, as the former may not fully replicate the functions of the latter. Whether ATII cells express ORs to detect environmental factors and whether ORs like OR51B5 have protective or harmful effects remain to be investigated in the future.

Intriguingly, evident differences manifest in how farnesol exerts effects between animal models and diverse cell types. This discrepancy primarily stems from the current inadequacy of research on ORs in COPD, particularly the scarcity of animal studies, which potentially yield contradictory findings. Current evidence suggests OR effects exhibit cell-type specificity: Farnesol activates OR51B5, reducing viability in bronchial epithelial cells (39) yet accelerating the regeneration rate of the HaCaT cell and keratinocyte monolayers (59). Additionally, the M2OR database (https://m2or.chemsensim.fr/) reveals that farnesol serves as a ligand for multiple ORs beyond OR51B5, including OR10S1, OR2A1, OR2A7, and notably OR1A2. Relevant studies confirm that activation of OR1A2 on human alveolar macrophages suppresses secretion of pro-inflammatory cytokines CXCL-8 and IL-6 (18). Moreover, the Farnesoid X Receptor functions as another farnesol receptor, demonstrating anti-inflammatory effects in A549 cells (63). Therefore, we attribute the discrepancies between cellular and animal models primarily to the exclusive focus on OR51B5 in human bronchial epithelial cells in cellular studies, whereas systemic administration of farnesol in animal models lacks targeting specificity, potentially activating multiple receptors across diverse cell types and consequently generating divergent effects.

#### 4.4.2 Airway remodeling: inflammation-driven structural reprogramming

Airway remodeling in COPD represents a dynamic inflammatory-driven restructuring process, with pathological crosstalk between epithelial cells and smooth muscle cells constituting its core mechanism. Chronically inflamed epithelial cells secrete mediators like IL-8 and IL-6, amplifying local inflammation while disrupting epithelial barrier integrity and basement membrane structure. These alterations create permissive conditions for abnormal extracellular matrix (ECM) deposition. Concurrently, inflammatory mediators induce smooth muscle hypercontractility via paracrine signaling, contributing to airflow limitation. They also drive pathological smooth muscle hypertrophy and hyperplasia, progressively thickening airway walls (64). While these mechanisms establish the foundation for structural remodeling, their precise regulatory networks remain incompletely mapped. As previously discussed, ORs influence both inflammatory initiation and structural remodeling cascades. Emerging evidence positions ORs as modulators of epithelial-mesenchymal interactions and ECM metabolism, suggesting therapeutic potential for interrupting progressive airway wall thickening.

**Figure 3:**
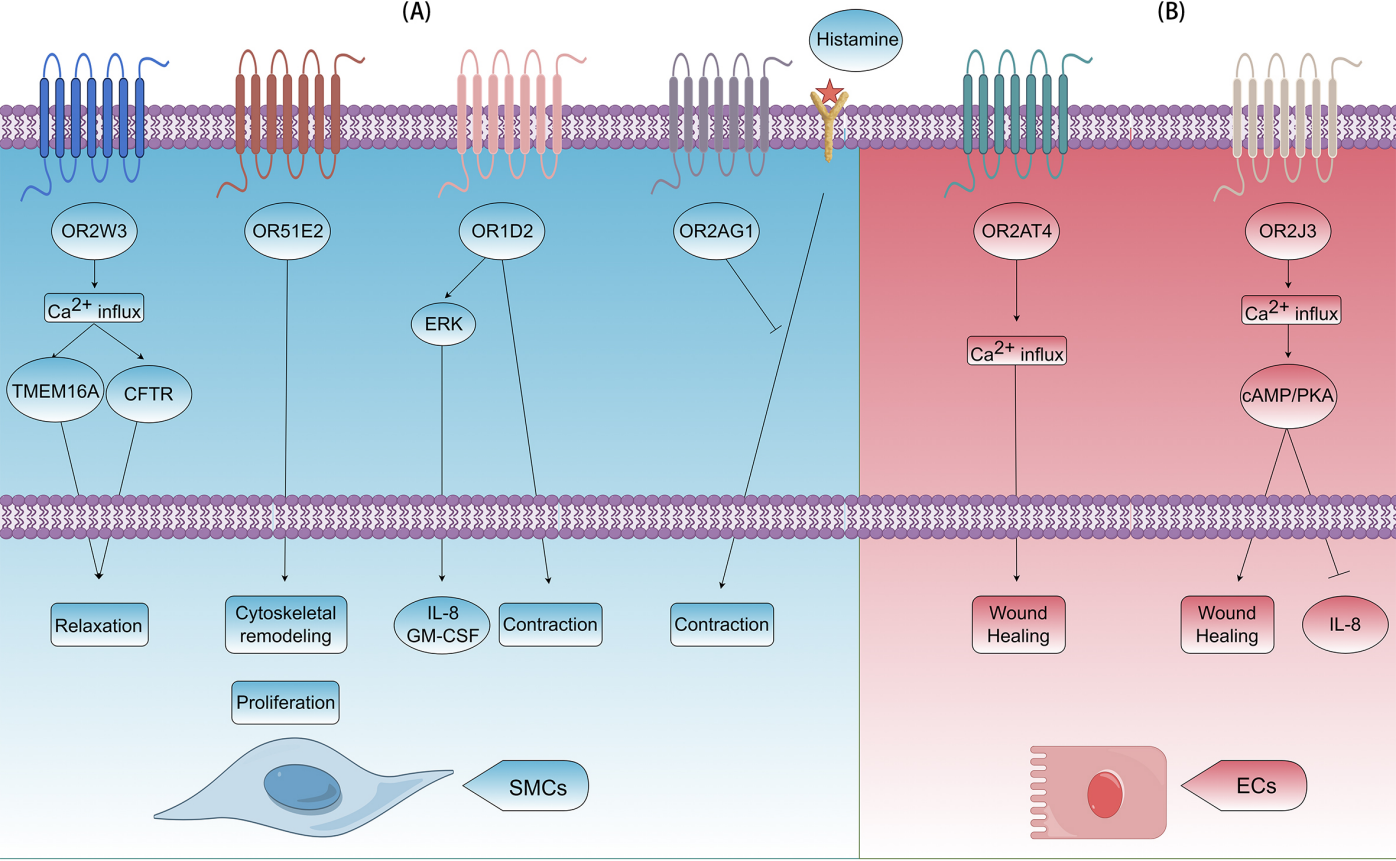
Effects of ORs on airway remodeling in COPD. **(A)** In airway smooth muscle cells (SMCs), OR2W3, OR51E2, OR1D2, and OR2AG1 can be activated. OR2W3 induces relaxation by Ca^2+^ influx and activating TMEM16A and CFTR; OR51E2 induces cytoskeletal remodeling and cell proliferation; OR1D2 induces contraction and release of IL-8/GM-CSF by ERK signaling pathway; OR2AG1 induces contraction by blocking histamine pathway. **(B)** In airway ECs, OR2AT4 and OR2J3 can be activated. OR2AT4 promotes wound healing by Ca^2+^ influx; OR2J3 promotes wound healing and inhibits release of IL-8 by Ca^2+^ influx and the cAMP/PKA pathway.

Airway smooth muscle cells precisely regulate bronchial diameter through contraction-relaxation cycles, maintaining airway patency and modulating airflow resistance. In COPD pathogenesis, pathological hyperplasia (cell proliferation) and hypertrophy (cell enlargement) of these cells significantly contribute to fixed airflow limitation by thickening airway walls and reducing luminal diameter. Smooth muscle-expressed ORs demonstrate exquisite environmental sensitivity, modulating airway tone through multiple signaling cascades. For instance, monoterpene nerol activates OR2W3, which triggers Ca^2+^ influx and then co-activates TMEM16A and CFTR, ultimately leading to smooth muscle relaxation and bronchodilation (6). This unique mechanism presents a potential new target for bronchodilator development. Short-chain fatty acids (SCFAs), which are gut microbiota metabolites, can reach the lungs via the bloodstream and may influence airway inflammation and remodeling (65, 66). In isolated HASMCs, acetate and propionate activate OR51E2, regulating cytoskeletal remodeling and inhibiting cell proliferation (5). However, in other cell types, activation of OR51E2 by β-ionone can activate the ERK1/2 pathway via the Gβγ-PI3Kγ-ARF1 pathway on the Golgi apparatus (67). Activation of this pathway can promote cell proliferation (68), potentially inducing airway remodeling and exacerbating COPD. Beyond airway smooth muscle, studies on Olfr78 (murine ortholog of human OR51E2) reveal that propionate induces vasodilation by activating this receptor and Gpr41 in vascular smooth muscle cells (69). Notably, lactate serves as an additional Olfr78 ligand (27). During hypoxia, accumulated lactate may exert dual effects: stimulating Olfr78 in carotid body glomus cells to enhance ventilation (70), while potentially relaxing bronchial smooth muscle cells to improve oxygenation and tissue perfusion. The OR family is extremely large, and different ORs can exert opposing effects on cell functions. For example, activation of OR1D2 by its ligand bourgeonal promotes HASMCs contraction, while activation of OR2AG1 by amyl butyrate inhibits histamine-induced contraction (19). Meanwhile, OR1D2 activation enhances the secretion of inflammatory factors, such as IL-8 and GM-CSF, through the ERK pathway, which is closely linked to airway remodeling (**Figure 3A**).

Airway epithelial cells serve as central orchestrators in COPD pathogenesis, contributing to both chronic inflammation initiation and structural remodeling progression. OR51B5 activation in human dermal fibroblasts promotes collagen secretion (7), and a similar mechanism may occur in bronchial epithelial cells, potentially contributing to the collagen deposition associated with airway remodeling in COPD. Moreover, OR2AT4 and OR2J3 are functionally expressed in human bronchial epithelial cells. OR2AT4 activation by Brahmanol promotes airway epithelial wound healing, akin to its role in skin and scalp repair (40). However, this activation can also induce cytoskeletal remodeling and reduce cell-to-cell connection integrity in human keratinocytes, highlighting its complex role in tissue homeostasis (71). When stimulated by cinnamaldehyde, OR2J3 inhibits IL-8 secretion and reduces cell proliferation without affecting cell viability (40), presenting a potential therapeutic target (**Figure 3B**). Conversely, Helional activates OR2J3 on A549 cells, triggering PI3K pathway activation, which leads to apoptosis and proliferation inhibition (72), highlighting the differential effects of ligands on the same receptor in different cell types.

#### 4.4.3 Other related mechanisms

COPD progression involves multifaceted mechanisms, with chronic inflammation and associated airway remodeling constituting primary drivers. Other pathological components include pulmonary vascular remodeling, genetic predisposition, and protease/antiprotease imbalance, each contributing to disease heterogeneity. The potential role of olfactory receptors in these processes remains unclear, but their involvement can represent an underexplored avenue for understanding and treating COPD.

As a risk factor of COPD, exposure to indoor VOCs has been shown to impair lung function in mice. Specifically, VOCs exposure induces PANoptosis in mouse pulmonary microvascular endothelial cells (MPVECs), characterized by elevated levels of Mlkl, Caspase3, and Gsdmd (73). Subsequently, these cells secrete Gas6, which binds to Axl on fibroblasts, promoting their transformation into myofibroblasts. This process may contribute to pulmonary fibrosis, a hallmark of COPD progression. Moreover, Lyral, a common spice allergen (74), activates OR10J5, which is highly expressed in vascular endothelial cells. The activation increases intracellular calcium concentration, then activates the AKT and ERK phosphorylation pathways, and promotes vascular endothelial cell migration and angiogenesis (75). Olfr603, another olfactory receptor expressed in mouse vascular endothelial cells, awaits further investigation to elucidate its specific function (76).

Genetic variations in OR2AG2 are significantly associated with the development of asthma (77). Given that asthma is a recognized risk factor for COPD, it is reasonable to postulate that genetic variations in ORs can also influence an individual’s susceptibility to COPD. Elucidating these genetic associations could pave the way for the development of personalized medicine strategies for COPD prevention and treatment.

The neuroendocrine system also plays a role in the relationship between ORs and COPD. PNECs, although present in a small proportion within the lung tissue, may play a significant role in airway regulation. In COPD patients, the number of PNECs is increased, and some of these cells express OR2W1 and OR2F1 (45). Activation of these receptors by nonanal and citronellal leads to a decrease in serotonin levels within PNECs and the release of the neuropeptide calcitonin gene-related peptide (CGRP). CGRP and serotonin can then act on adjacent epithelial cells, smooth muscle cells, and other cell types, potentially modulating airway tone, mucus secretion, and the inflammatory response. The potential of PNECs as a novel chemosensory cell type offers new perspectives for developing targeted therapies for airway-related diseases like COPD and asthma.

In addition to the aforementioned mechanisms, other mechanisms like protease/antiprotease imbalance, mucus hypersecretion, and cellular senescence also play significant roles in the pathogenesis and progression of COPD. However, the relationship between ORs and these mechanisms remains unexplored, which warrants further investigation in future studies.

## 5 Future directions

### 5.1 ORs in pulmonary microbial communities and gut-lung axis communication

Accumulating evidence have highlighted the crucial role of the lung microbiota in both healthy and disease states (78–81). In healthy individuals, the lungs host a diverse microbiota, primarily composed of Prevotella, Streptococcus, Veillonella, Fusobacterium and Haemophilus (79–81). However, in disease conditions such as COPD, the microbiota balance is disrupted, characterized by a shift in the composition and abundance of bacteria. In asthmatic patients, the lung microbiota is dominated by Haemophilus, Moraxella and Neisseriaceae, with notable increases of Haemophilus, Staphylococcus, Pseudomonas and Actinomyces (82–84). In COPD patients, Pseudomonas, Streptococcus, Prevotella and Haemophilus are the predominant components, with a marked increase in Pseudomonas aeruginosa, Lactobacillus, Proteobacteria and Haemophilus (85–87). Bacterial components and their metabolites can act as ligands for ORs (88, 89). For instance, diaminopimelic acid, a component of the cell wall of gram-negative bacteria (90), induces the release of MCP-1 in mouse lung macrophages, exacerbating lung inflammation (47). Additionally, 12 types of VOCs, including heptane and methylated cycloalkanes, were identified and they are generated by cultures of bacteria and viruses associated with respiratory infections (43). How these microorganisms affect the occurrence and progression of COPD, and how cells in the lung tissue sense these factors that may induce lung injury remain to be further explored. ORs may be one of the potential mechanisms. Future research should focus on elucidating the complex interplay between the lung microbiota, ORs, and COPD development, which may open new avenues for therapeutic interventions targeting the microbiota-OR axis.

The gut-lung axis represents a bidirectional communication system between the gut microbiota and the respiratory system. SCFAs, produced by the fermentation of dietary fibers by gut microbiota (91), can reach the lungs via the bloodstream and modulate pulmonary inflammatory responses (65, 92–95). Given that many SCFAs are agonists or antagonists of ORs (5, 69, 96), ORs may play a key role in facilitating communication within the gut-lung axis. Understanding how ORs respond to SCFAs in the context of the gut-lung axis could provide insights into the development of novel therapeutic strategies for COPD. For example, modulating the gut microbiota to increase the production of beneficial SCFAs or developing drugs that target specific ORs activated by SCFAs may offer innovative approaches to regulate airway inflammation and remodeling in COPD patients. However, further research is required to clarify the precise mechanisms by which ORs mediate the effects of SCFAs on the respiratory system.

### 5.2 Treatment targeting ORs

Approximately 40% of available drugs target GPCRs (97). As the largest subgroup within the GPCRs family, ORs hold significant promise for drug development. Recognizing ORs as potential regulators of pulmonary inflammatory diseases such as COPD, provides new opportunities for therapeutic intervention. Synthetic OR agonists and antagonists can be designed to selectively modulate the mechanisms in COPD, potentially reducing inflammation, preventing airway remodeling and improving lung function.

However, several challenges must be addressed before OR-based therapies can be translated into clinical practice. Among these challenges, one major obstacle is the lack of receptor-ligand specificity. ORs can interact with multiple ligands, and ligands can bind to multiple receptors. Besides, Conventional small-molecule agonists/antagonists often cause off-target effects due to the widespread expression of ORs in extrapulmonary tissues (e.g., heart and kidneys). Ensuring the specificity of receptor-ligand interaction is crucial to minimize off-target effects. Comprehensive mapping of OR ligand profiles and the development of targeted drug delivery systems are essential to overcome this challenge. On one hand, we should continue developing agonists/antagonists with higher specificity. Current studies have confirmed that Corilagin attenuates atherosclerosis by inhibiting Olfr2 signaling (98). On the other hand, we can develop ORs-related nanomaterials to enhance tissue targeting, cell targeting, and receptor targeting. Meanwhile, future research should also focus on understanding the precise distribution of ORs in different cell types and tissues within the lung, mapping the complex signal transduction networks they are involved in, and elucidating their functions in the COPD pathophysiology.

Nevertheless, despite advances in single-cell RNA sequencing and spatial transcriptomics, detecting low-abundance ORs remains challenging due to their minimal transcript levels. We look forward to the availability of more sensitive high-throughput sequencing methods in the future to aid in the identification of specific expression of ORs in the lungs, new OR ligands and the development of more effective drugs. These efforts may not only expand our understanding of the olfactory system but also facilitate the development of innovative treatment strategies for COPD and other respiratory diseases.

## 6 Summary

A comprehensive review of current ORs research underscores their capacity to modulate diverse cellular signaling pathways, positioning them as promising targets for early diagnostic and therapeutic strategies in COPD. While traditionally associated with olfaction, ORs have now been identified as pivotal regulators in respiratory diseases. Despite this significance, in-depth studies on ORs specifically in COPD remain scarce; existing research primarily focuses on mechanistic links to phenotypes like chronic inflammation and airway remodeling. Nevertheless, evidence emerging from these limited investigations suggests a critical role for ORs in COPD treatment, particularly for symptom management.

Moreover, translating basic research discoveries into clinical applications remains a significant challenge, necessitating further investigation. Key barriers include tissue-specific delivery inefficiencies and ligand-receptor interaction complexities, which require innovative solutions to bridge the gap between mechanistic insights and therapeutic implementation. Future investigations should integrate single-cell omics with artificial intelligence (AI) to construct multidimensional “receptor-ligand-phenotype” maps, enabling personalized therapeutic strategies. Concurrent development of pH-responsive nanocarriers for airway-targeted drug delivery, combined with gut microbiome modulation, promises to overcome current limitations in anti-inflammatory therapy resistance. Breakthroughs in OR research not only redefine the role of GPCRs in respiratory diseases but also drive a paradigm shift from symptom management to causal disease interception. These advances establish transformative pathways to reduce COPD-related global disability rates and healthcare burdens through mechanism-driven precision medicine.
